# Visceral leishmaniasis in northwest China from 2004 to 2018: a spatio-temporal analysis

**DOI:** 10.1186/s40249-020-00782-4

**Published:** 2020-12-03

**Authors:** Canjun Zheng, Liping Wang, Yi Li, Xiao-Nong Zhou

**Affiliations:** 1 Chinese Center for Diseases Control and Prevention (China CDC), Beijing, 102206 China; 2 State Key Laboratory of Severe Weather & Key Laboratory of Atmospheric Chemistry of CMA, Chinese Academy of Meteorological Sciences, Beijing, 100081 China; 3 Chinese Center for Diseases Control and Prevention, National Institute of Parasitic Diseases, Shanghai, 200025 China

**Keywords:** Visceral leishmaniasis, Spatio-temporal analysis, Spatial regression, China

## Abstract

**Background:**

Although visceral leishmaniasis (VL), a disease caused by parasites, is controlled in most provinces in China, it is still a serious public health problem and remains fundamentally uncontrolled in some northwest provinces and autonomous regions. The objective of this study is to explore the spatial and temporal characteristics of VL in Sichuan Province, Gansu Province and Xinjiang Uygur Autonomous Region in China from 2004 to 2018 and to identify the risk areas for VL transmission.

**Methods:**

Spatiotemporal models were applied to explore the spatio-temporal distribution characteristics of VL and the association between VL and meteorological factors in western China from 2004 to 2018. Geographic information of patients from the National Diseases Reporting Information System operated by the Chinese Center for Disease Control and Prevention was defined according to the address code from the surveillance data.

**Results:**

During our study period, nearly 90% of cases occurred in some counties in three western regions (Sichuan Province, Gansu Province and Xinjiang Uygur Autonomous Region), and a significant spatial clustering pattern was observed. With our spatiotemporal model, the transmission risk, autoregressive risk and epidemic risk of these counties during our study period were also well predicted. The number of VL cases in three regions of western China concentrated on a few of counties. VL in Kashi Prefecture, Xinjiang Uygur Autonomous Region is still serious prevalent, and integrated control measures must be taken in different endemic areas.

**Conclusions:**

The number of VL cases in three regions of western China concentrated on a few of counties. VL in Kashi Prefecture, Xinjiang Uygur Autonomous Region is still serious prevalent, and integrated control measures must be taken in different endemic areas. Our findings will strengthen the VL control programme in China.

## Background

Visceral leishmaniasis (VL) is a parasitic disease that is endemic in more than 62 countries, with an estimated 1 300 000 new patients each year worldwide [1]. VL has been one of important public health problems in the People’s Republic of China. Prior to the initiation of a national control program in 1951, there were about 530 000 VL cases in China in 1951 [2–4].

The first confirmed case of VL in China was reported in 1907 [5]. In the following years, cases have been reported in 16 provinces north of the Yangtze River, which were recognized as endemic areas [6]. The disease was especially rampant in eastern China and also prevalent in Shaanxi, Gansu, and Xinjiang in western China; sporadic cases occurred in Beijing, Liaoning, Ningxia, Qinghai, Inner Mongolia, Hubei, Shanxi, and the southern part of Sichuan. According to surveys conducted in 1951 [3, 7, 8], about 530 000 cases were reported and the average incidence in different provinces was 10–50 per 10 000 people. Following great efforts of the national control program, the disease has been largely brought under control in the eastern regions of the country after the 1960s [6, 9, 10]. VL cases decreased from more than 530 000 originally distributed in more than 660 counties to several hundred cases concentrated in less than 30 counties in the new century.

However, transmission was not interrupted in the mountainous and desert regions, where sporadic cases continue to present. Currently, VL is still prevalent or sporadically distributed in six western provinces and autonomous regions, including Xinjiang, Gansu, Sichuan, Shaanxi, Shanxi, and Inner Mongolia. Four species of sandfly (*Ph. Chinensis, Ph. Longiductus, Ph. Wui and Ph. Alexandri*), which serve as staple vectors for the transmission of VL in China, are distributed in different geographic areas, contributing to the different ecological features and transmission patterns in different areas [3, 11]. The most sensitive population threatened by VL in western China is children. The main parasite species, *Leishmania infantum*, which mainly affects children, is distributed in some remote areas of the regions, and new cases are reported every year. Among these endemic regions, three provinces/autonomous regions, i.e., Xinjiang, Gansu, and Sichuan, still face serious health problems due to VL transmission, and infants are the main population under threat of infection [7]. In China, there are two major protozoan parasites, i.e., *Leishmania donovani* and *L. infantum*, cause VL. They are transmitted by three species of sandfly, i.e., *Phlebotomus longiductus*, *Phlebotomus wui*, and *Phlebotomus chinensis* [3, 11]. Two epidemiological types of VL, i.e., the anthroponotic type and the zoonotic type, have been classified based on the ecosystem and source of infection in China [12].

The anthroponotic type, caused by *L. donovani*, currently is endemic in the oases of the plains of Kashi prefecture, Xinjiang Uygur Autonomous Region. Most cases infected with *L. donovani* occur in young people and children. The transmission cycle is from human to human, and no animal host has been found yet. Its vector is a peridomestic species, *P. longiductus*, which is widely distributed in Xinjiang. During history, the control strategies carried out in endemic areas for the anthroponotic type of VL, which was also widespread in eastern China in the 1950s, emphasized on mass screening for patients, in combination with vector control. A large number of infected patients were found and received medical treatment in the 1950s. At the same time, the density and natural infection rate of sandflies gradually decreased by indoor or outdoor use of insecticides. As a result, the prevalence of the anthroponotic type of VL was effectively controlled by the end of the 1950s in China.

The zoonotic type, caused by *L. infantum*, involves an animal host as the principal source of infection [7, 13]. This type has been divided into two subtypes, the mountainous subtype and the desert subtype, based on the ecosystem and epidemiological characteristics, i.e., geographical and landscape characteristics, vector (sandfly) species and their ecology, and source of infection [14, 15]. Contrarily to anthroponotic VL, spraying of insecticides had was little effect on vector control because of the exophilic behavior of the sandfly of zoonotic endemic areas. In mountain subtype endemic areas, killing infected dogs and prohibition of raising domestic dogs, combined with spraying non-infected dogs with deltamethrin, markedly interrupted the transmission of mountain subtype VL. The various control strategies for different types of VL made great contributions to the control of VL, and by the end of the 1950s, it was almost eliminated in China.

Unfortunately, there was no effective way to control desert subtype VL due to the unknown source of infection and wild habitats of the vector; spraying of insecticide showed little effect [12]. People who live in houses of poor quality usually lack individual protection and consciousness, increasing the risk of being bitten by sandflies and becoming infected. Moreover, it should be considered that most cases reported after year 2000 were of this type of VL. The most prominent endemic region of desert subtype VL is the oases of the plains in Kashi Prefecture, Xinjiang Uygur Autonomous Region. Three spatio-temporal clusters with respect to the distribution of VL-infected families during 1990–2005 were identified by scan statistical analysis [16, 17]: (i) Boshikelam, Haohan township of Kashi city and Awati town in Shufu county, (ii) reclaimed farmland of Bachu county andand (iii) Yinwusitang town in Shufu county and Yangdaman town in Shule county.

Besides Xinjiang, VL is still endemic in some mountainous areas in north Sichuan Province and south Gansu Province. Infected dogs and patients with *L. donovani* are major infection sources. Although the disease has been under control since the end of the 1950s, it has gradually reemerged since 1972 [7, 18, 19] in north Sichuan and south Gansu. After that, control strategies, including treatment of patients, health education, dog registration and management, and sandfly control in combination with sanitary policies of cities or towns, were implemented, and the numbers of reported cases in those counties decreased significantly. However, VL cases still occur sporadically.

During our study period (2004–2018), more than half of cases reported in China occurred in the Xinjiang Uygur Autonomous Region, and effective control measures should be taken there. Moreover, there were two outbreaks in Jiashi county of Xinjiang (a desert subtype of the zoonotic type endemic area), during 2008–2009 and 2014–2015, respectively [20–22], with the incidence rate more than 20 times as high as the average annual incidence. It is still unclear whether some kinds of animals transmit the pathogen there. To elucidate the reasons behind the outbreaks, researchers have explored several factors, including land cover [23], meteorological factors [20], and socio-economic status [24], suggesting some possible associations between outbreak and air temperature, relative humidity, land use, and socio-economic status. In addition, several studies [6–9, 11–13] on VL epidemiology have been performed in China, including a retrospective review of notified VL cases between 2005 and 2010 based on the passive surveillance data, a description of epidemiological features of VL in China during 2004–2012 [12, 13], and a review of phlebotomine sandflies transmitting VL and their geographical distribution in China [11, 14]. All those studies have shown that cluster VL transmission patterns have been observed in some areas of western China in different periods of time, indicating that intervention strategies should focus their attention on high-risk transmission areas.

The objective of this study is (i) to explore the spatial and temporal characteristics of VL in Sichuan Province, Gansu Province and Xinjiang Uygur Autonomous Region in China from 2004 to 2018 and (ii) to identify the risk areas for VL transmission, which could provide evidence-based information for effective control efforts. We performed an investigation in a spatio-temporal manner using an additive–multiplicative decomposition of the conditional intensity function [25].

## Materials and methods

### Data source

The surveillance and population data were obtained from the National Diseases Reporting Information System (NDRIS) operated by the Chinese Center for Disease Control and Prevention. In China, cases of VL, one of the notifiable infectious diseases, have been compulsorily reported through the NDRIS according to the National Regulation on the Control of Communicable Diseases (Version 2005). Each VL case record included a range of information on variables such as name, age, gender, diagnosis, date of birth, date of onset, and current address code, among other factors.

Daily meteorological data covering the whole country from 2004 to 2018, including mean/minimum/maximum air temperature, relative humidity, precipitation, and mean/minimum/maximum land surface temperature, were obtained from the China Meteorological Administration.

Since the parasites, *Leishmania* spp., have a digenetic lifecycle, alternating between a mammalian host and insect vectors (phlebotomine sandflies), we simulated the two types of models to understand the transmission of VL, including a spatial regression model in the vector and a spatio-temporal model in humans.

### Spatial autocorrelation analysis

We used R Language [25] to calculate and plot Moran’s *I* to evaluate the spatial pattern of VL cases. Moran’s *I* is a measure of spatial autocorrelation characterized by a correlation in a signal among nearby locations in space. Moran’s *I* is defined as.

$${\text{I}}=\frac{N}{\sum i\sum j{\omega }_{ij}}.\frac{\sum i\sum j{\omega }_{ij}\left({x}_{i}-\stackrel{-}{X}\right)\left({x}_{j}-\stackrel{-}{X}\right)}{\sum i({x}_{i}-\stackrel{-}{X}{)}^{2}},$$

where *N* is the number of spatial units indexed by *i* and *j*; X is the variable of interest; $\stackrel{-}{X}$ is the mean of X; and ${\omega }_{ij}$ is an element of a matrix of spatial weights.

### Spatial regression

We applied a spatial regression model with R Language [25] to explore the impacts of meteorological factors on VL transmission. This model is expressed by the following equation:

$$Y = WY + X\nu + \varepsilon ,$$

where *Y* is the variable representing the dependent variable; *W* is a matrix of spatial weights; *WY*, which expresses the spatial dependence in *Y*, is a new explanatory variable added to the regression model that represents the weighted average of the dependent variable in neighboring areas; *W* is the spatial autoregressive parameter, such that if *W* = 0, there is no autocorrelation and the model becomes equivalent to the usual linear regression model; *X* is the matrix with the values of the independent variables; *ν* is one of the regression parameters; and *ε* represents the random independent errors with zero mean and constant variance. The best-fitting model was further verified using the Akaike information criterion (AIC).

### Spatio-temporal two-component conditional intensity function model

In this study, we applied a spatio-temporal model by Held and Paul [26, 27] to study the spatial and temporal characteristics of the VL surveillance data. This is an additive–multiplicative model for the conditional intensity function (CIF) of an infectious disease process continuous in space–time with events occurring in a prespecified observation period [0, *T*] (*T* > 0). In this model, disease counts *Y*_*it*_ in regions *i* (*i* = 1,... *I*) and period *t* (*t* = 1,... *T*) are assumed to follow a negative binomial distribution:

$${Y}_{it}|\mathrm{Y}\sim \mathrm{NegBin}\left({\mu }_{it},\varphi \right), i=1,\dots I, t=1, \dots ,T,$$

with additively decomposed mean,

1 $${\mu }_{it}={v}_{it}{e}_{it}+{\lambda }_{it}{Y}_{i,t-1}+{\phi }_{it}\sum_{j\ne 1}{\omega }_{ij}{Y}_{j,t-1}.$$

If *φ* = 0, the binomial distribution changes to a Poisson distribution. In (1), ${v}_{it}{e}_{it}$ represents the endemic component, reflecting the local risk factors; ${\lambda }_{it}{Y}_{i,t-1}$ and ${\phi }_{it}\sum_{j\ne 1}{\omega }_{ij}{Y}_{j,t-1}$ are observation-driven epidemic components: an autoregression on the number of cases at the previous time point and a “spatio-temporal” component capturing transmission from other units, that is, epidemic risk.

Each of ${v}_{it}$, ${\lambda }_{it}$, and ${\phi }_{it}$ is a log-linear predictor of the form

$$\mathrm{log}\left({*}_{it}\right)={\alpha }^{(*)}+{b}_{i}^{(*)}+{\beta }^{(*)}{z}_{it}^{(*)},$$

where “*” represents one of *ν*, *ϕ*, or *λ*, containing fixed and region-specific intercepts as well as effects of exogenous covariates ${z}_{it}^{(*)}$ including time effects.

## Results

### Data description

During the 15 years from 2004 to 2018, a total of 4896 VL cases from 435 counties in 29 provinces/municipalities/autonomous regions were reported in China. Of those cases, 89.3% (4374/4896) occurred in three provinces/autonomous regions, namely, Xinjiang, Gansu, and Sichuan (Table 1). Most cases were reported in Xinjiang Autonomous Region (43.1%, *n* = 2109) followed by Gansu Province (32.6%, *n* = 1595) and Sichuan Province (13.7%, *n* = 670). These three regions are geographically connected. The trend of the whole country and that of Xinjiang were very similar, because most cases were reported in Xinjiang (Additional file 1, Fig. 1). Also, the trend of Sichuan was similar to that of Gansu. Therefore, we discuss (i) Xinjiang and (ii) Sichuan and Gansu separately.

**Table 1:** Number of visceral leishmaniasis cases reported in China during 2004 and 2018

Year	Xinjiang	Gansu	Sichuan	Other area	Total	%^a^
2004	13	3	9	2	27	92.6
2005	161	93	57	22	333	93.4
2006	127	114	48	15	304	95.1
2007	121	153	77	23	374	93.9
2008	323	160	50	28	561	95.0
2009	281	157	60	27	525	94.9
2010	138	155	64	26	383	93.2
2011	54	162	69	24	309	92.2
2012	43	112	52	22	229	90.4
2013	23	80	40	25	168	85.1
2014	157	89	30	36	312	88.5
2015	409	53	23	46	531	91.3
2016	198	84	34	57	373	84.7
2017	45	105	35	75	260	71.2
2018	16	75	22	94	207	54.6

^a^Percentage of the three provinces

Table 2 shows the mean or sum value of meteorological factors in our study area during our study period.

**Table 2:** Description of the meteorological factors in the study area during 2004 and 2018

	Xinjiang	Gansu	Sichuan
Relative humidity^a^	52.16	65.73	82.63
Mean temperature^a^	17.24	14.65	8.40
Precipitation^b^	47.88	410.79	1777.17
Land surface temperature^a^	63.30	32.14	32.42

^a^Mean value

^b^Sum value

### Spatial autocorrelation results

**Fig. 1:**
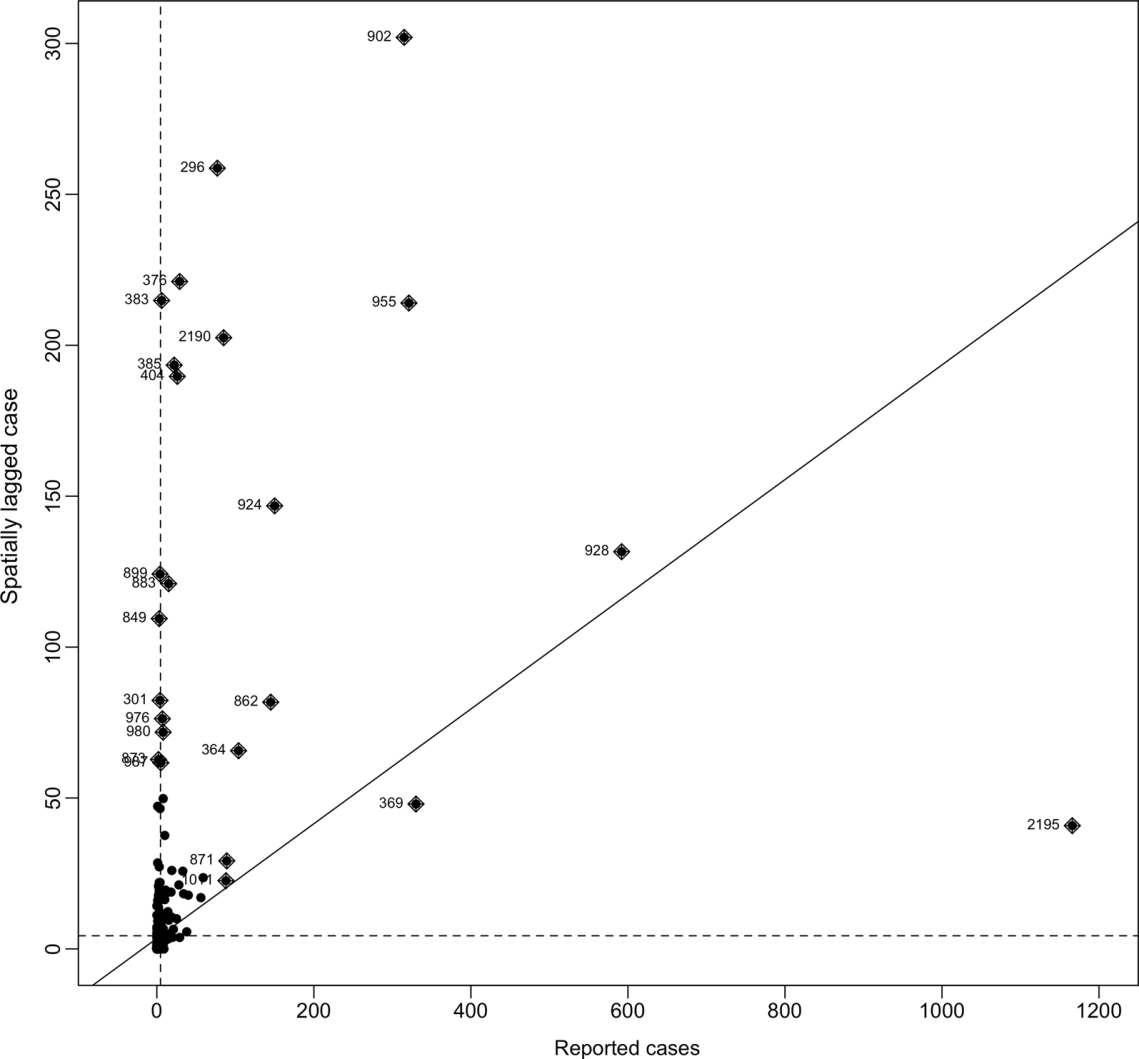
Moran’s plot of visceral leishmaniasis cases in four quadrants. Plots in the first quadrant mean high observation value and short distance. Plots in the second quadrant suggest middle observation value and they are near those counties with high observation values

It could be seen that county 2195 and county 369 in Xinjiang were in the first quadrant, and these two adjacent counties had the highest two number of cases. Counties with codes 902, 955, 924, 862, 364, 2190, and 1011 in the second quadrant had fewer cases than those in the first quadrant, but were near or adjacent to them, as Fig. 1 shows. Also, we could see that most counties were in the third quadrant, which means these counties had the fewest cases and most of them were adjacent. The observed observed Moran’s *I* was 0.18, with a *P*-value < 0.01 suggesting clustering (or dispersion) probably occurring in a few counties.

Plots in the first quadrant mean a high observation value and a short distance. Plots in the second quadrant suggest a medium observation value and they are near those counties with high observation values.

### Spatial regression between meteorological factors and VL cases

The spatial regression analysis (Table 3) shows that during our study period, mean air temperature had a significant positive association with VL incidence (*P* < 0.01). No significant associations between precipitation, relative humidity, and VL cases were observed.

**Table 3:** Spatial regression of visceral leishmaniasis incidence and precipitation, air temperature and relative humidity during 2004–2018

Variables	Estimate	Std. error	*P*-value
Intercept	−0.37	4.45	0.94
Precipitation	−0.05	0.089	0.57
Air temperature	0.16	0.034	3.51E−06
Relative humidity	−0.028	0.046	0.54

### Spatio-temporal  results of Sichuan and Gansu

**Fig. 2:**
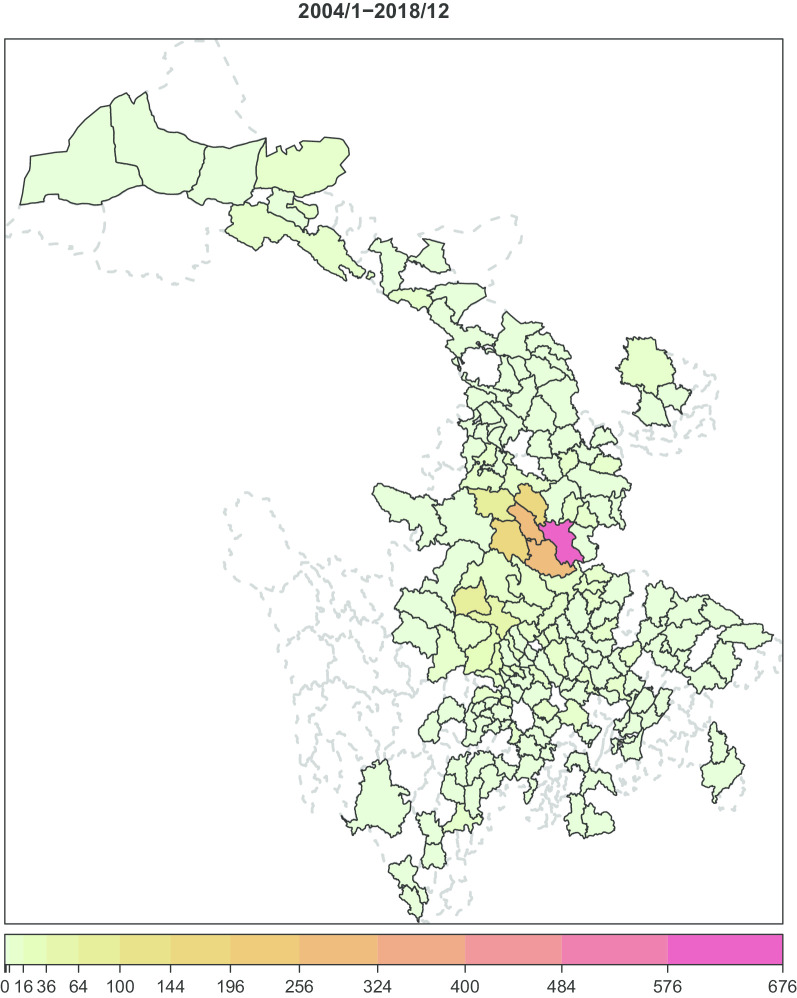
Spatial visualization of the time aggregated visceral leishmaniasis data in Sichuan and Gansu

**Fig. 3:**
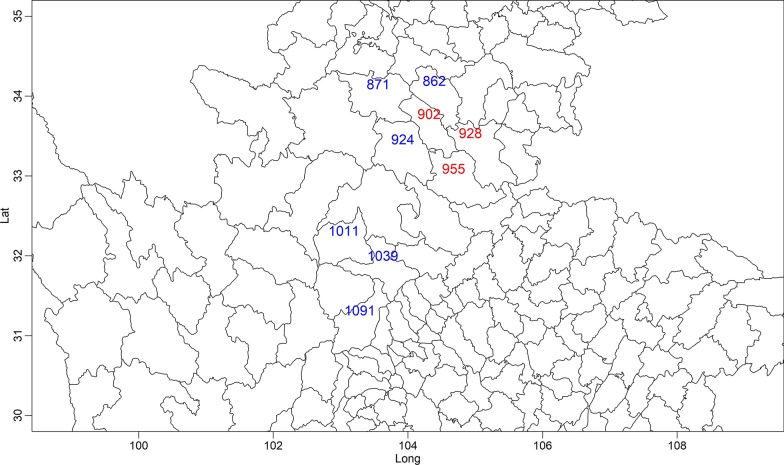
Geographic positions of the 9 counties with more than 50 accumulative cases

Figure 2 shows the pattern of time aggregated VL cases in Sichuan and Gansu, and nine counties with more than 50 aggregated cases are marked in Fig. 3.

**Fig. 4:**
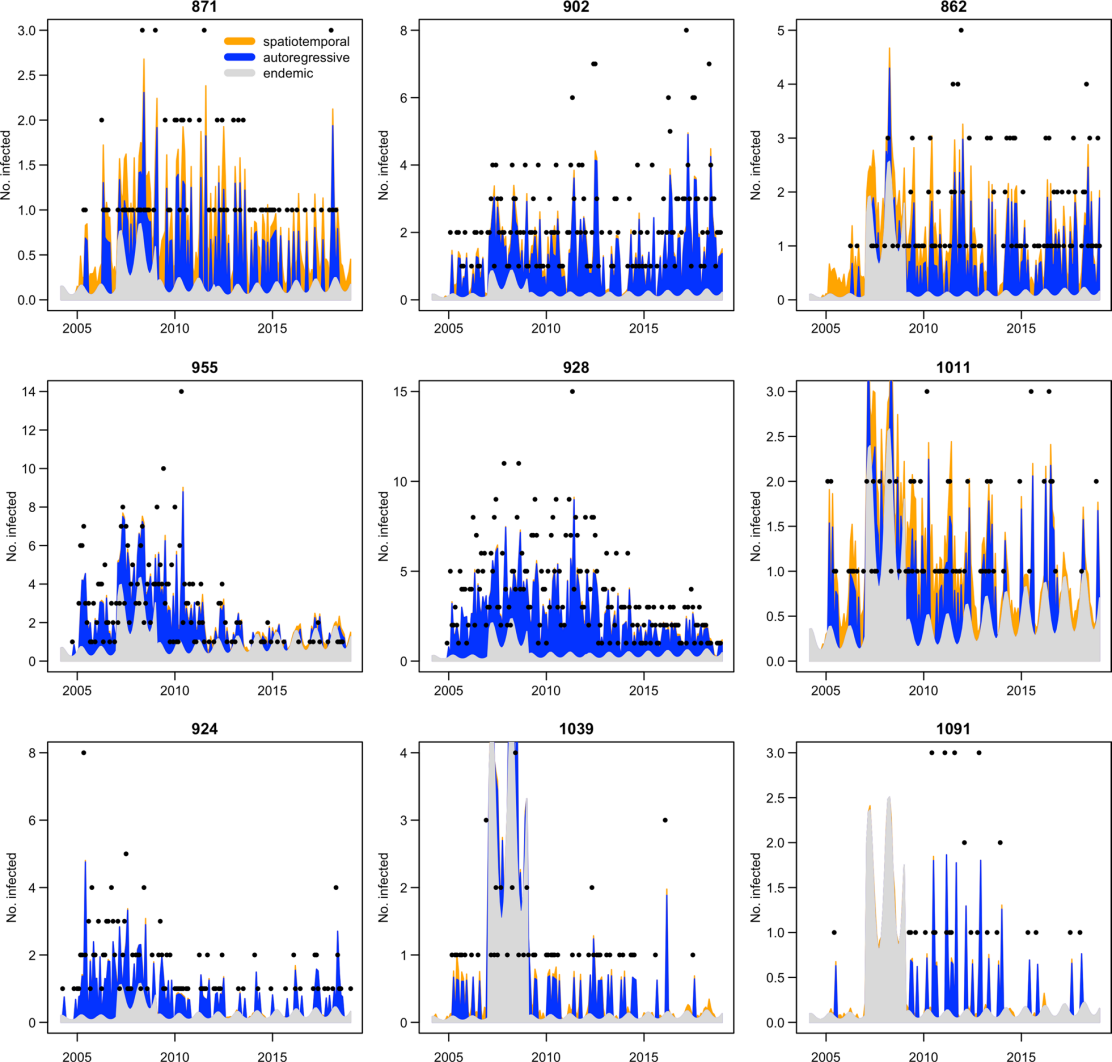
Time series and the decomposed components (endemic, autoregressive and epidemic) of the 9 counties during our study period

It could be seen that these counties are geographically connected and most cases were reported in counties 928, 955, and 902 (in red). In Fig. 4, the decomposed VL risk components (endemic component, epidemic component, and autoregressive component) of these counties are presented. It could be seen that counties 955, 924, and 902 were the three counties that had most cases and they had different time series characteristics. Cases in county 902 had two peaks, in 2010–2012 and 2016–2018. Cases in county 955 focused before 2011, peaked in 2010, and few cases were reported after 2012. Cases reported in county 928 peaked in 2011, and also, most cased were reported before 2012.

Considering their geographic positions, in county 928, the autoregressive component had the highest value, and the value of the epidemic component was much lower compared, suggesting the local risk was more important, and it was not much affected by the neighbor county. For county 955, the endemic component and the epidemic component had high values, and the number of reported cases decreased significantly after 2011. After 2011, the epidemic component of county 955 increased, suggesting a spatial interaction effect between county 928 and 924. County 902 also had a relatively high epidemic component, with a relative averaged number of cases each year during our study period. For county 924, the endemic component was the largest, and most cases were reported before 2010.

For Gansu and Sichuan, it could be seen that three counties in south Gansu had the highest number of cases, and the endemic component had the highest value. The other three counties in Sichuan (1011, 1039, and 1091), with 88, 59, and 34 reported cases, respectively, had the highest epidemic component, suggesting that they were affected by the northern area. Almost half of the cases reported in counties 1039 and 1091 were imported, and in county 1011, which is the nearest to Gansu, more than 90% of cases were imported.

### Spatio-temporal results of Xinjiang

**Fig. 5:**
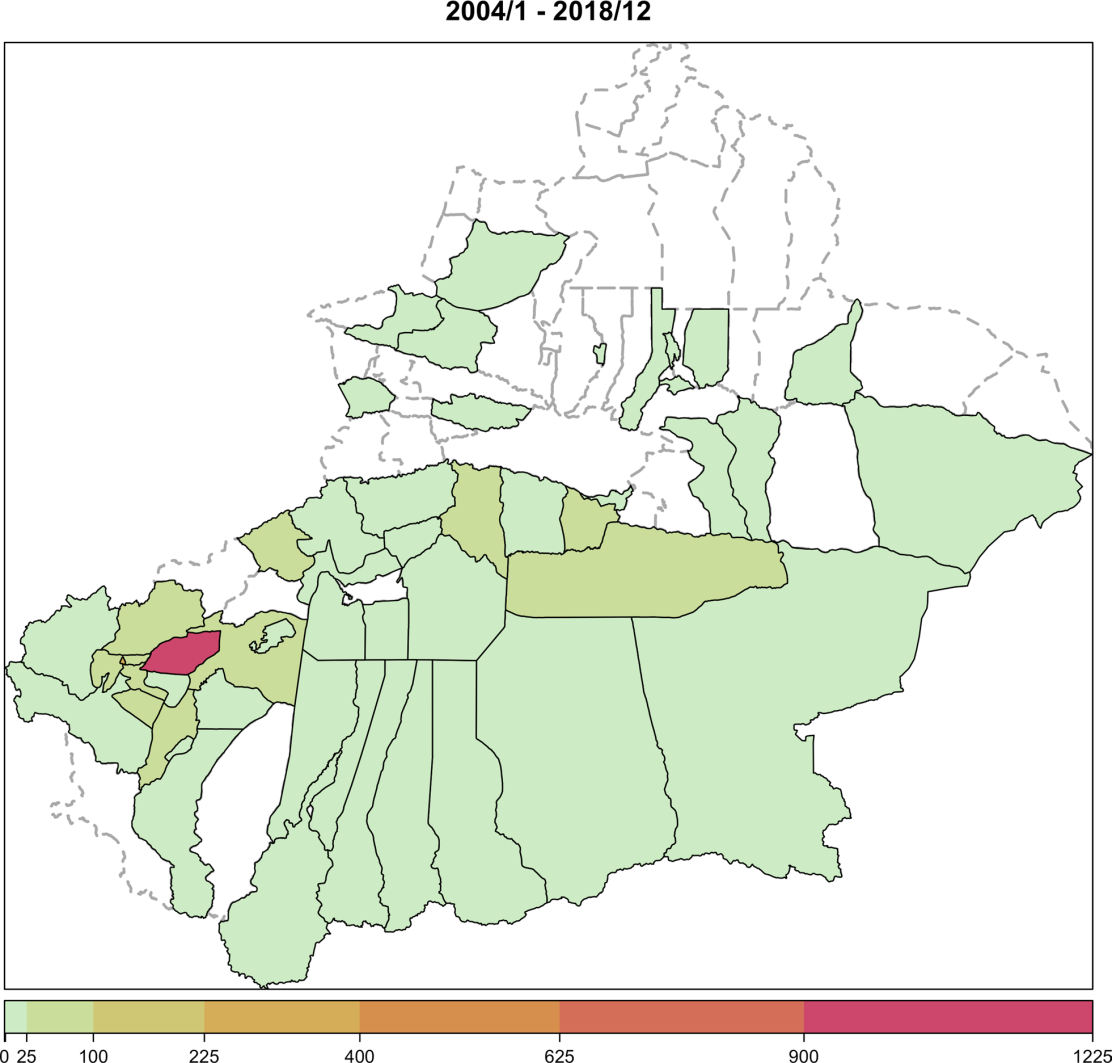
Spatial visualization of the time aggregated visceral leishmaniasis data for each county in Xinjiang

**Fig. 6:**
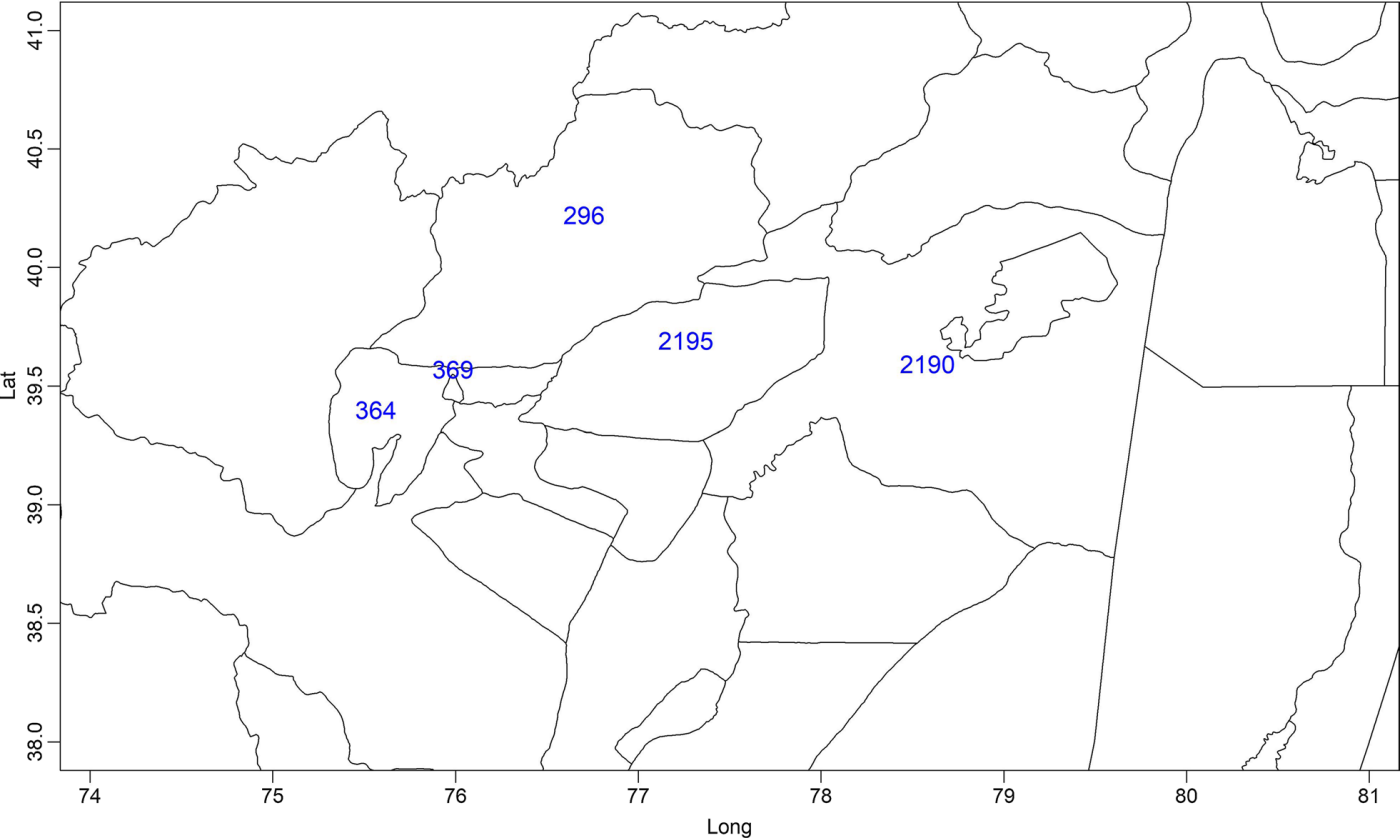
The geographic positions of the five counties with communicated cases more than 50 in Xinjiang

Spatial visualization of the time aggregated visceral leishmaniasis data for each county in Xinjiang was shown in Fig. 5, and the geographic position of the five counties where more than 50 cases were reported during our study period was marked in Fig. 6. It could be seen that the number of reported cases among these counties varied very much: county 2195 has the highest number of cases (1166), which was much higher than that of the second highest county (369).

**Fig. 7:**
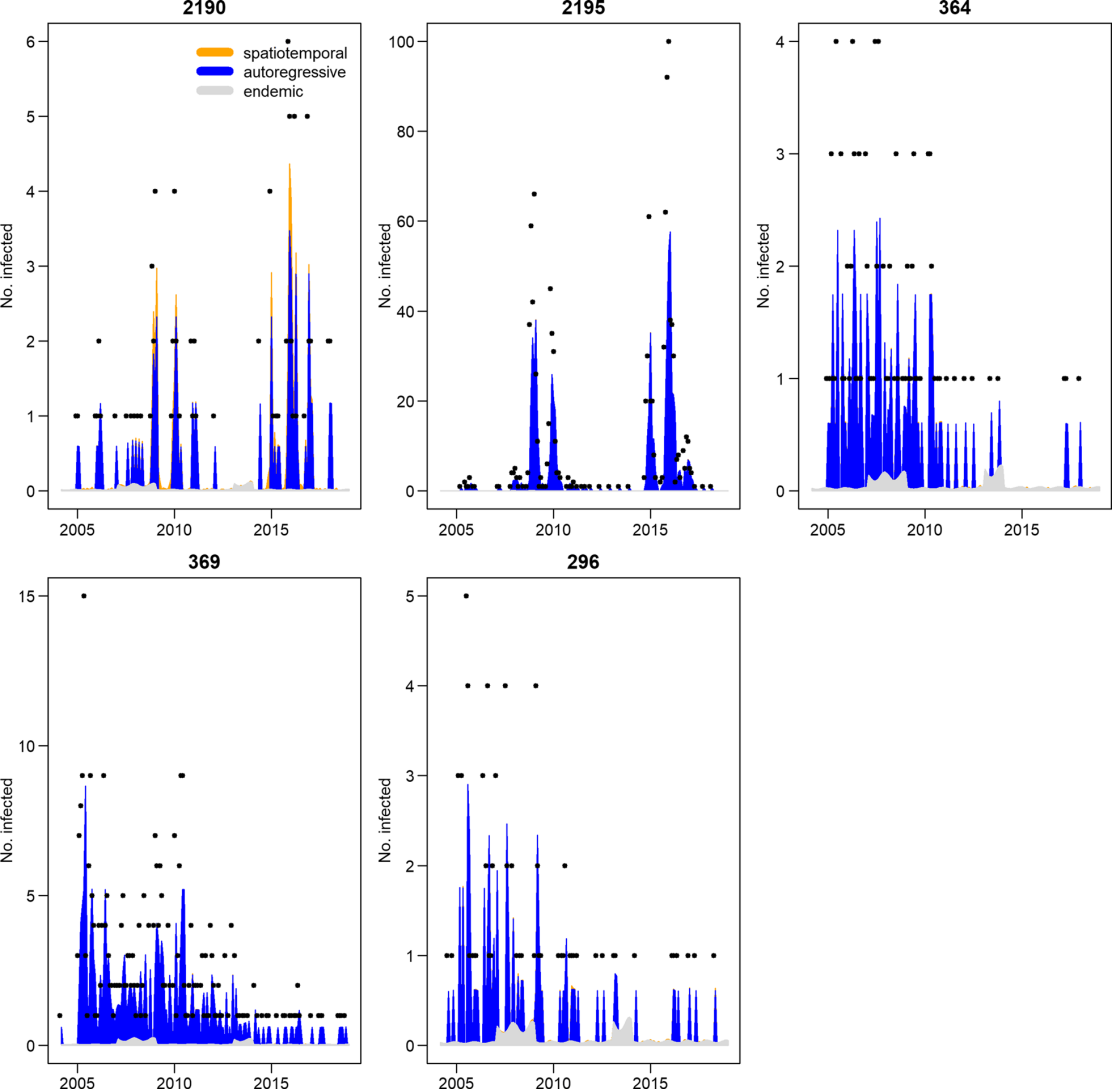
Decomposed components (endemic, autoregressive and epidemic) for the 5 districts with more than 50 cases

As shown in Fig. 7, county 2195 had a very low epidemic component and the endemic component was the largest, suggesting it was very little affected by neighbor counties. Counties 369 and 296 showed similar characteristics: the endemic component was the largest. The epidemic component of county 2190, which is directly adjacent to county 2195, was high, showing the spatial interaction between neighbor counties. Table 4 shows the ratio of imported cases; it can be seen that all cases in county 2190 were imported, and most cases in county 364 were imported.

**Table 4:** Number of aggregated and imported cases and the value of three components in our study area

Study code	Number of aggregated cases	Number of imported cases	Ratio of case importation (%)	Autoregressive component	Epidemic component	Endemic component
1091	34	16	47.1	0.69	1.34	0.58
1039	59	27	45.8	0.59	1.38	0.55
924	173	37	21.4	1.26	0.86	2.22
1011	94	92	97.9	0.48	0.6	0.41
928	648	58	9.0	2.54	0.66	0.47
955	353	182	51.6	1.98	2.21	3.16
862	147	129	87.8	1.18	0.88	0.45
902	325	194	59.7	1.49	1.35	4.06
871	95	55	57.9	0.46	0.6	1.28
364	113	93	78.8	0.95	3.08	2.13
296	93	28	30.1	0.31	0.18	2.29
369	330	88	26.7	1.12	0.13	3.21
2195	1166	16	1.4	1.62	−0.19	3.86
2190	89	89	100	0.17	1.24	2.09

In Xinjiang, more than 60% of cases were reported in county 2195, where two outbreaks occurred between 2007 and 2015; 35% of cases in Xinjiang were reported in four counties adjacent to county 2195, suggesting very significant endemic characteristics.

## Discussion

In this study, we explored the spatio-temporal characteristics of VL in three provinces/autonomous regions of west China. We found significant spatial aggregation of the prevalence during our study period: most cases were concentrated in 14 counties, indicating that the transmission of VL in local epidemics or small-scale outbreaks is still ongoing. Among these counties, five are in Xinjiang and nine are in Gansu or Sichuan. For the three counties in north Sichuan (counties 1011, 1039, and 1091), spatial interaction was the main component, and more than half of the cases reported were imported. For the five counties in Gansu, it could be seen that spatial interaction and local risk were both important. VL in Gansu and Sichuan was mostly zoonotic, and the cases showed spatial aggregation. This might be related to the distribution of local infectious sources, vectors, and terrain characteristics.

For the five counties in Xinjiang, significant spatial difference was found. County 2195, which is located in the middle of the five counties and had the largest number of cases, had a significant spatial effect on county 2190, which is adjacent to on its eastern border. Age distribution and month reported of these two counties were very similar, and were very different from the other three counties (see Additional files 1, 2, 3). There might be two different communication modes in these five counties, which might be due to different species of leishmaniasis, vectors, or others factors [8, 10, 11]. Further studies are required to improve our understanding of these factors. In county 2195, it is important to spray insecticide before September and in county 2190, it important to prevent imported risk. Anthroponotic VL near the Kashgar oasis in Xinjiang is susceptible to large-scale epidemics, which happened in Shandong Province and Henan Province (eastern China). However, anthroponotic VL in Xinjiang showed small-scale spatial aggregation. Moreover, these five counties are all not in the list of known anthroponotic VL areas. Therefore, it is important to explore why the distribution of these vectors is limited to the Kashgar oasis and to find out if there is a specific distribution of peridomestic vectors (*P. longiductus*) (Additional file 4).

Our model revealed important spatial characteristics of VL in Xinjiang, Gansu, and Sichuan. The results showed that local risk was the main factor in some counties, suggesting that vectors and animal sources of transmission have existed for a long time. In fact, these counties are all located in underdeveloped areas, where the local government did not take this issue seriously and almost no funds were reserved for disease prevention. Moreover, vectors in these areas are habituated by wildlife, and thus spraying with pesticides indoor has very limited effects. Thus, for these areas where local risk was the main factor, the government should investigate the habitats and distribution of disease vectors and animal sources of infection. Based on the investigation, prevention and control strategies should be adopted. Meanwhile, measures including screening sources of infection, killing infected dogs, and restricting dog keeping should also be taken. Moreover, health policies should be strengthened to local residents and medicated mosquito nets should be used to reduce sandfly bites.

Our results also showed that in some counties (like counties 1011 and 862 in Sichuan and Gansu and county 2190 in Xinjiang), more than half of the cases reported were imported from neighbor counties, suggesting a spatial effect (epidemic) was the main risk. Although the population in western China is relatively sparse and those areas are less developed, there is still frequent and close-up population mobility. Imported cases might induce a larger epidemic area. It is needed to popularize health education to tell local citizens not to travel to surrounding epidemic areas. In the meantime, personal protection measures should be taken to prevent sandfly bites, and medical treatment should be sought as soon as possible after the onset of disease. At the same time, the local disease prevention and control department should assess the risk of local transmission caused by imported cases and control the further spread of VL.

We also explored the effects of meteorological factors on VL risk. In this study, we found significant associations between ambient temperature and VL risks in Jiashi County, Xinjiang Uygur Autonomous Region and this is in accordance with another study of ours [20]. This suggests an important role of meteorological factors in the process of VL infection. Similar to other sandfly borne infectious diseases, air temperature affects VL by affecting the vectors. The growth and reproduction of sandflies in western China generally occurs from May to October, and reaches its peak in summer from July to August. In this study, we found a significant positive relationship between the disease incidence and mean air temperature. Thus, the higher the air temperature, the higher the disease prevalence/the risk of disease. Hence, it is important to spray pesticides to vectors’ habitats before breeding season; during its transmission season, the government should guide residents in epidemic areas to use sandfly nets, especially insecticide impregnated nets and sandfly repellent incense, reduce outdoor activities, and prevent vector sandflies from biting.

At the global level, VL is still a serious problem in some countries, including Brazil, Argentina, Germany, and Spain [28–31]. Because of the differences in meteorological factors and natural environments, the types of endemic areas, the transmission risk of VL, and the underlying transmission determinants are also quite different among these countries [32–37].

There are some limitations of our study. First of all, there lack information about sandflies like the distribution, density, seasonal characteristics and how sandflies infecting humanbeings. Second, knowledge about the habits of sandflies is still very limited. In future study, we would focus on the investigation on the vectors.add a paragrah to highlight limitations of the study.

## Conclusions

During our study period, nearly 90% of cases occurred in some counties in three western provinces/autonomous regions (Xinjiang, Sichuan and Gansu), and a pattern of significant spatial clustering observed. The transmission risk, autoregressive risk and epidemic risk of these counties during our study period were also well predicted.

The number of VL cases in three regions of western China concentrated on a few of counties. VL in Kashi Prefecture, Xinjiang Uygur Autonomous Region is still serious prevalent, and integrated control measures must be taken in different endemic areas. The results from the present study indicate that the number of VL cases in three regions of west China concentrated on several counties. VL in Kashi Prefecture, Xinjiang Uygur Autonomous Region is still serious and differential control measures must be taken in different endemic areas. Our findings will strengthen the VL control programme in China.

## Supplementary information

**Additional file 1: Figure S1.** Age distribution of cases reported in the five counties of Xinjiang.

**Additional file 2: Figure S2.** Age distribution of imported cases in the five counties of Xinjiang.

**Additional file 3: Figure S3.** Month of case reported distribution of imported cases in the five counties of Xinjiang.

**Additional file 4: Table S1.** Code and name of the investigated counties.

## Data Availability

All data generated or analyzed during this study are kept confidential by China CDC and Chinese Academy of Meteorological Scieces. The datasets are available from the corresponding author on a reasonable request.

## Abbreviation

VL: Visceral leishmaniasis
NDRIS: National Diseases Reporting Information System
