# Supplementary figures and images for: Visceral leishmaniasis in northwest China from 2004 to 2018: a spatio-temporal analysis
Source: Infect Dis Poverty. 2020 Dec 3;9:165. doi: 10.1186/s40249-020-00782-4 (PMC7713028; doi:10.1186/s40249-020-00782-4)

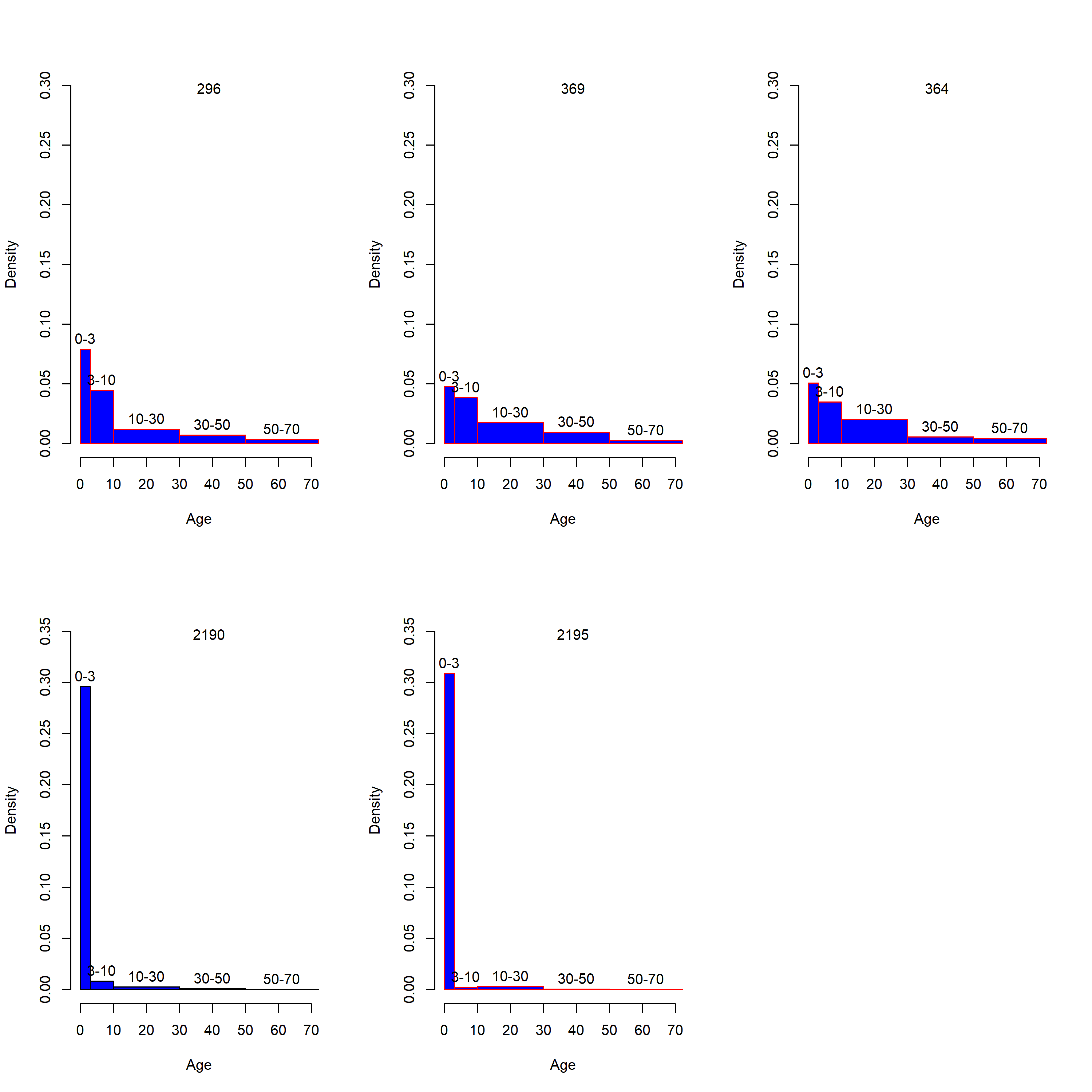

Supplement: Supplementary file 1 — Additional file 1: Figure S1. Age distribution of cases reported in the five counties of Xinjiang. [file 40249_2020_782_MOESM1_ESM.png]

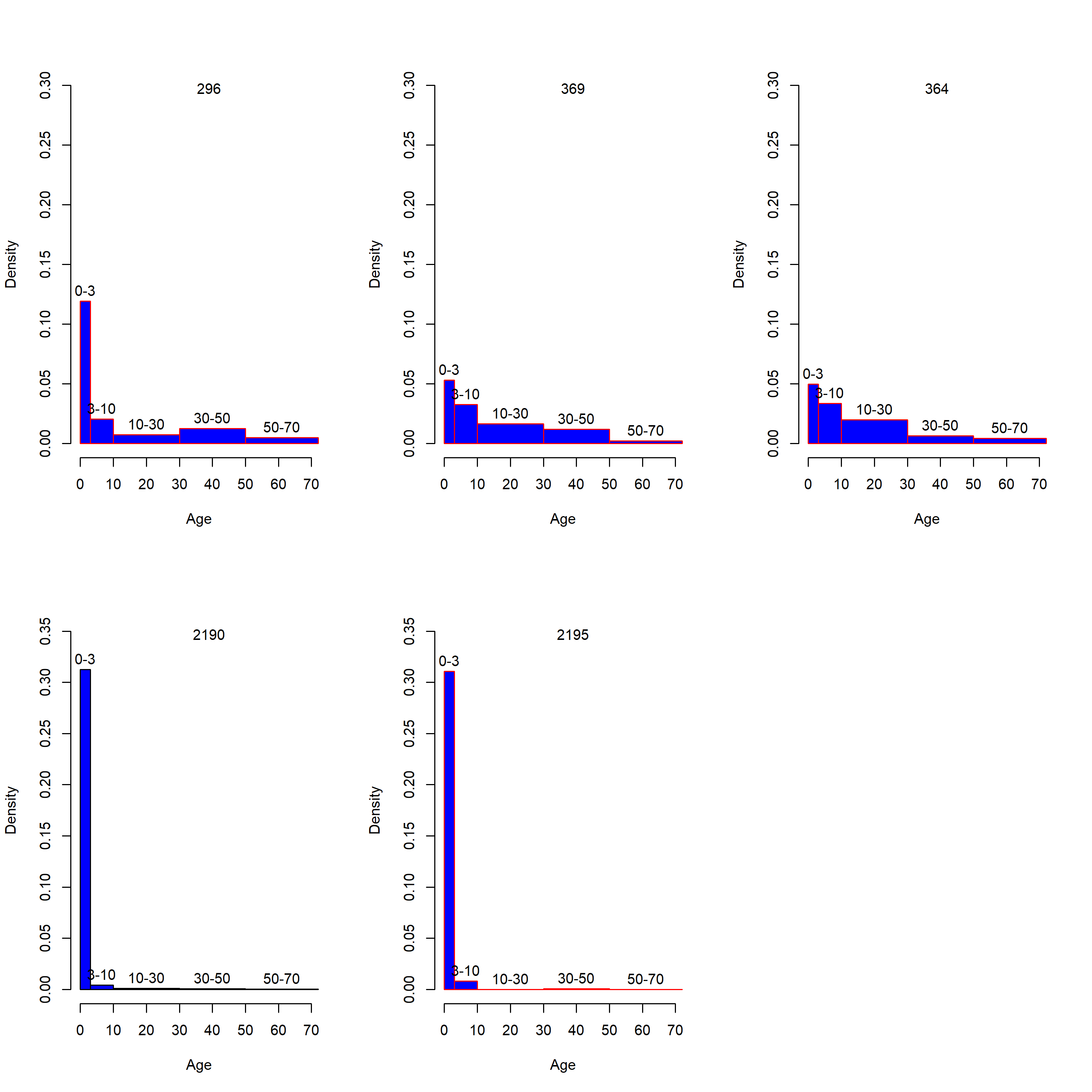

Supplement: Supplementary file 2 — Additional file 2: Figure S2. Age distribution of imported cases in the five counties of Xinjiang. [file 40249_2020_782_MOESM2_ESM.png]

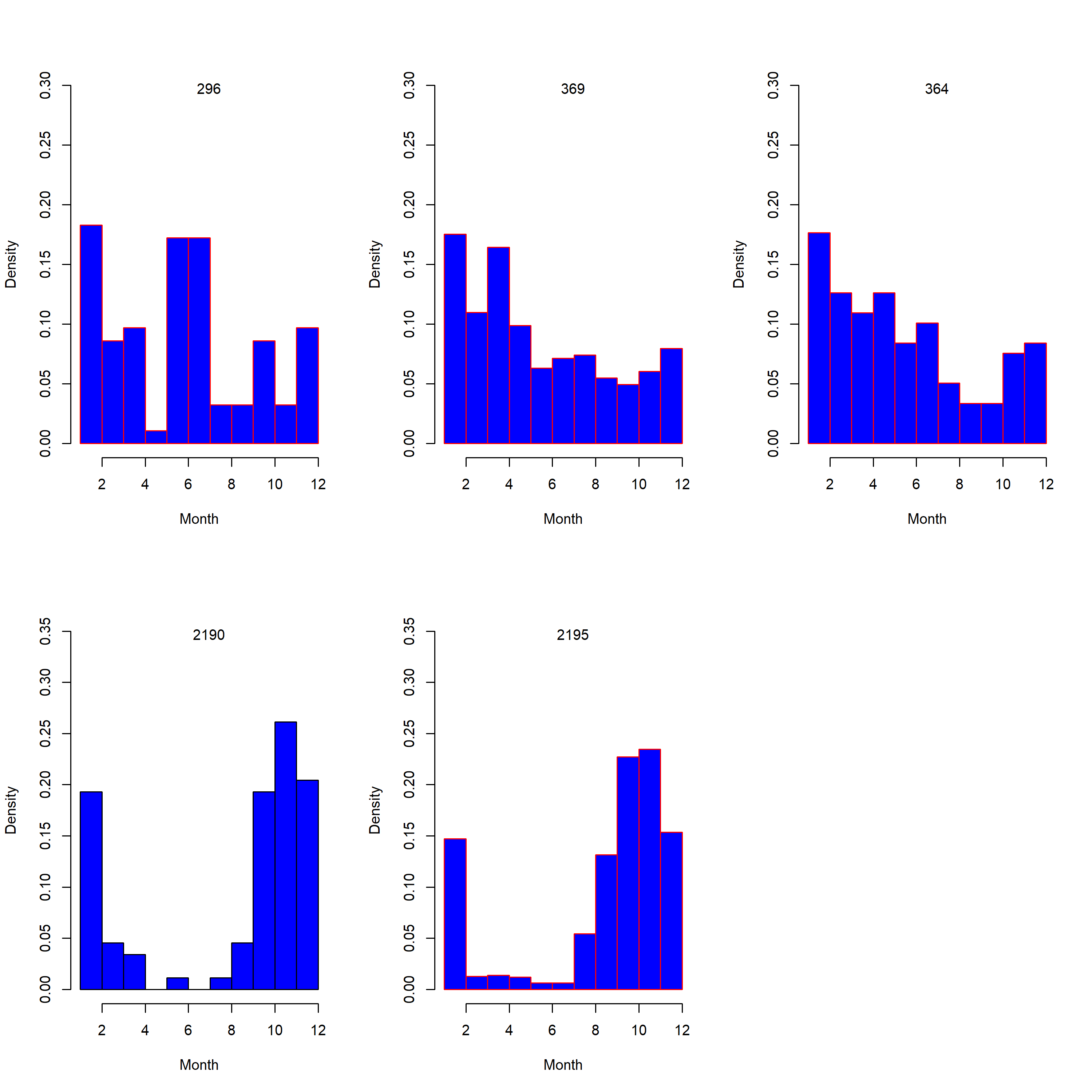

Supplement: Supplementary file 3 — Additional file 3: Figure S3. Month of case reported distribution of imported cases in the five counties of Xinjiang. [file 40249_2020_782_MOESM3_ESM.png]
