# Supplementary material for: Visceral leishmaniasis in northwest China from 2004 to 2018: a spatio-temporal analysis
Source: Infect Dis Poverty. 2020 Dec 3;9:165. doi: 10.1186/s40249-020-00782-4 (PMC7713028; doi:10.1186/s40249-020-00782-4)
Supplement: Supplementary file 4 — Additional file 4: Table S1. Code and name of the investigated counties. [file 40249_2020_782_MOESM4_ESM.docx]

Table S1. Code and name of the investigated counties

| Code | Name | Longtitude | Latitude | Number of VL case |
| --- | --- | --- | --- | --- |
| 928 | Wudu | 33.388155 | 104.929866 | 592 |
| 955 | Wenxian | 32.942171 | 104.682448 | 321 |
| 902 | Zhouqu | 33.628181 | 104.3177 | 315 |
| 924 | Jiuzhaigou | 33.310997 | 103.92594 | 150 |
| 862 | Dangchang | 34.042655 | 104.394475 | 145 |
| 871 | Diebu | 34.00066 | 103.56087 | 89 |
| 1011 | Heishui | 32.165249 | 103.04836 | 88 |
| 1039 | Maoxian | 31.852222 | 103.62584 | 59 |
| 296 | Atushi | 40.132069 | 76.704468 | 77 |
| 369 | Kashi | 39.480732 | 75.987694 | 330 |
| 364 | Shufu | 39.392788 | 75.745979 | 104 |
| 2190 | Bachu | 39.585865 | 78.802711 | 85 |
| 2195 | Jiashi | 39.601994 | 77.260918 | 1166 |
